# Evaluating machine learning methodologies for identification of cancer driver genes

**DOI:** 10.1038/s41598-021-91656-8

**Published:** 2021-06-10

**Authors:** Sharaf J. Malebary, Yaser Daanial Khan

**Affiliations:** 1 Department of Information Technology, Faculty of Computing and Information Technology, King Abdulaziz University, P.O. Box 344, Rabigh, 21911 Saudi Arabia; 2 Department of Computer Science, School of Systems and Technology, University of Management and Technology, Lahore, Pakistan

**Keywords:** Biotechnology, Cancer, Computational biology and bioinformatics

## Abstract

Cancer is driven by distinctive sorts of changes and basic variations in genes. Recognizing cancer driver genes is basic for accurate oncological analysis. Numerous methodologies to distinguish and identify drivers presently exist, but efficient tools to combine and optimize them on huge datasets are few. Most strategies for prioritizing transformations depend basically on frequency-based criteria. Strategies are required to dependably prioritize organically dynamic driver changes over inert passengers in high-throughput sequencing cancer information sets. This study proposes a model namely PCDG-Pred which works as a utility capable of distinguishing cancer driver and passenger attributes of genes based on sequencing data. Keeping in view the significance of the cancer driver genes an efficient method is proposed to identify the cancer driver genes. Further, various validation techniques are applied at different levels to establish the effectiveness of the model and to obtain metrics like accuracy, Mathew’s correlation coefficient, sensitivity, and specificity. The results of the study strongly indicate that the proposed strategy provides a fundamental functional advantage over other existing strategies for cancer driver genes identification. Subsequently, careful experiments exhibit that the accuracy metrics obtained for self-consistency, independent set, and cross-validation tests are 91.08%., 87.26%, and 92.48% respectively.

## Introduction

A gene is a small area of a long DNA twofold helix particle, which comprises a direct arrangement of nucleotide sets. A gene is any area along with the DNA with information encoded that instructs a cell to deliver an item which generally is a protein. Each such protein is linked with some biological phenomenon that may and may not be physically apparent. DNA is the substance that shows up in strands. Each cell in an individual's body has a similar DNA, but each person's DNA is distinctive. Changes in genes referred to as mutations, play a vital role in the development or progression of cancer. Transformations can cause a cell to form proteins that influence how the cell develops^1^.

The human genome undergoes several genetic mutations and epigenetic changes. These changes are attributed to aging, heredity, and environmental factors. Exposure to carcinogenic mutagens can also be one of such environmental factors. Subsequent genetic mutations that unsettles the functional characteristics of a gene can ultimately lead to carcinogenesis. Change in functional characteristics of genes can cause an interruption in processes that regulate the natural balance between mitosis (replication of cells) and apoptosis (destruction of cells) causing the onset of cancer. Several mutations may occur within the genome but not all are carcinogenic. Studies show that functional alteration in genes that regulate cell growth and differentiation leads to cancer. Scientific data shows that very few of the mutations lead to cancer and a gene has to undergo several mutations before the onset of cancer. Cancer is one of the foremost advanced diseases that undermine human health. Cancer is driven by different sorts of genetic changes, for example, single nucleotide variations (SNVs), inclusions or erasures (Indels), and basic variations. The gene transformation that contributes to cancer tends to influence three fundamental sorts of genes—oncogenes, tumor suppressor genes, and DNA repair genes. Such genes that drive the development of cancer are called cancer driver genes. Contemporarily, passenger mutations do not cause the onset of cancer.

Not all genes are cancer driver genes neither do all mutations render a growth advantage to cancers. Very few of the mutations are cancerous. Subsequently, very few of the genes are cancer drivers. Based on the gene function, a gene may offer conducive circumstances for cancer growth, once mutated^2^. For instance, the function of the widely known TP53 gene is to encode the tumor suppressor p53 protein. DNA repair is supervised by the p53 protein within the cell. Certain mutations within the TP53 gene can compromise the ability of p53 to supervise these repairs increasing the risk of developing cancers. Tumor suppressor function of TP53 gene renders it as cancer driver once mutated. Hence by learning the gene characteristics it is possible to determine which gene can exhibit cancer driver traits. Genes are the heredity units containing protein-encoding information for a specific function. Computational intelligence algorithms are predominantly used for the identification of obscure patterns within genomic and proteomic data. This study endeavors to identify such patterns from cancer driver genes and decipher them against passenger genes.

A few major oncogenomic sequencing ventures, such as “The Cancer Genome Atlas” (TCGA)^3^, the “International Cancer Genome Consortium” (ICGC)^4^, and the “Therapeutically Applicable Research to Generate Effective Treatments” (TARGET), have made a comprehensive catalog of physical changes overall major cancer sorts^5,6^. Right now, numerous computational strategies have been proposed. Based on their fundamentals, existing strategies can be partitioned into different categories. The most typical kinds of strategies are based on transformation recurrence. These strategies discover “significantly mutated genes” (SMG) whose change rates are altogether higher than the foundation transformation rate and judge them as driver genes. These existing tools are mainly partitioned into three main categories i.e. frequency-based, sub-network methods, and hotspot-based methods by their basic rules.

Identification of cancer driver genes plays an important role in precision oncology and personalized cancer treatment. Scientists working on finding personalized treatment of cancer aims at identifying the genes involved in the onset of cancer and subsequently taking measures to silence those specific genes or their cancer-causing functional characteristics. Henceforth, a tool that could accurately and efficiently identify cancer driver genes is in profound demand. The ability to recognize such driver genes can enable us to disentangle the specific instrument of disease, and hence play a pivotal role in the advancement of research in novel medications and treatments for cancer. This study defines an assiduous methodology for a new prediction model for computational identification of cancer driver genes. The work adapts broadly used approaches in bioinformatics and computational science for the recognition of cancer driver genes^7–9^. A valuable and systematic sequence-based methodology for an organic framework can be planned by observing the following simple steps (1) development or determination of a substantial benchmark dataset for training and testing the prediction model; (2) definition of the organic arrangement tests with a viable numerical expression, reflecting their basic relationship with the targets concerned; (3) creating an effective computational algorithm for prediction; (4) validation of outcomes that equitably evaluate the expected precision (5) Providing a framework for public use based upon the carved out robust model.

## Materials and methods

**Figure 1:**
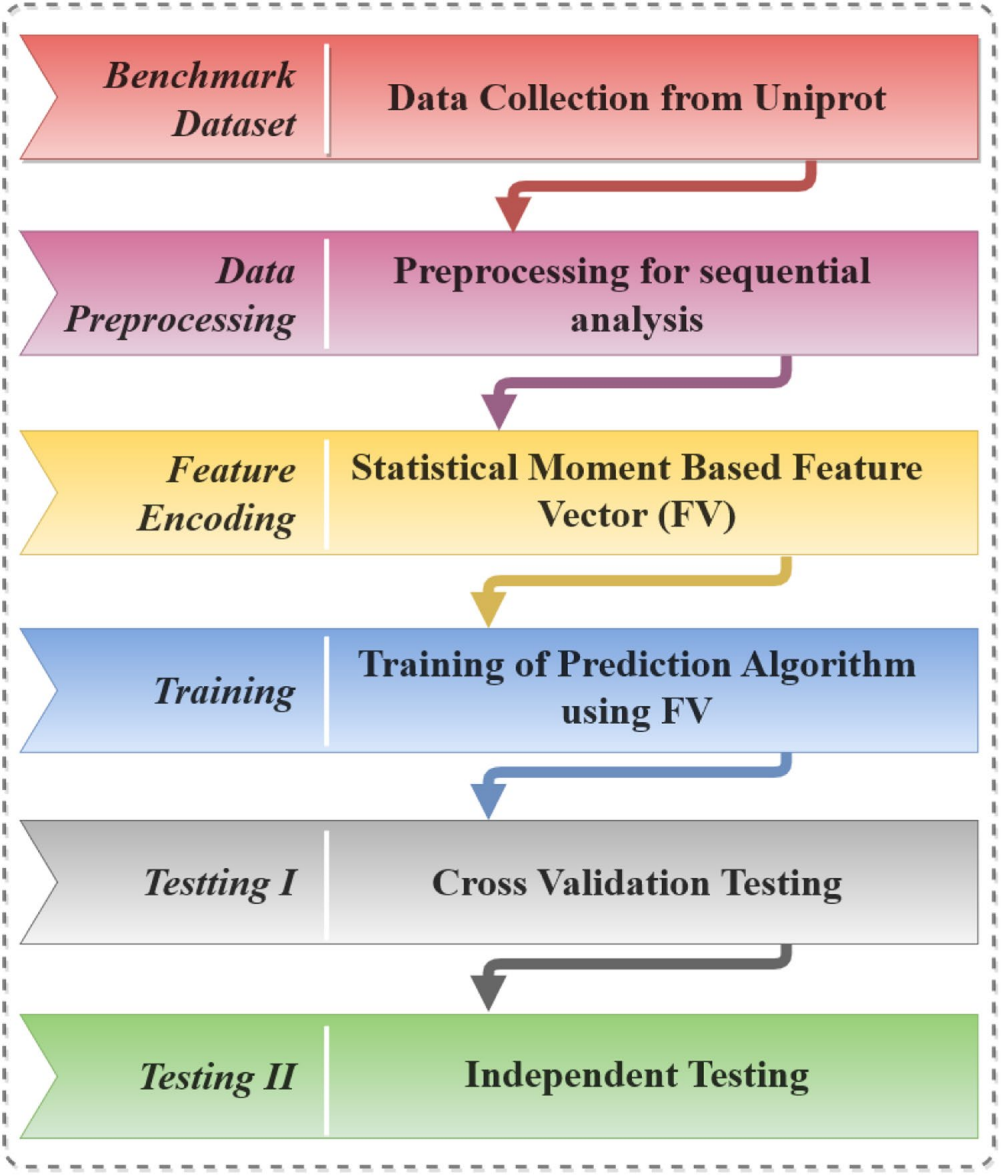
Steps towards the construction of a robust prediction model.

This section contains a detailed description and usage of the computational processes that run the show. The primary steps are selection/creation of benchmark dataset, test definitions, and extraction of feature vectors that highlight the attributes of the dataset while the final step is the development and training of a robust prediction model based on the material gathered within the feature vector as depicted in Fig. 1.

### Benchmark dataset collection and its preprocessing

The benchmark dataset usually comprises experimentally established unambiguous known samples. These samples are further used for training as well as testing purposes^10^. The purpose is to develop one high-quality benchmark dataset which is diverse, accurate, and relevant. Further, the outcome of the experimental work is substantiated through a range of experimental validation tests like independent set and subsampling (K-fold cross-validation) tests^11^. Coherent and meaningful data bears significance since the outcome received is a combination of numerous distinctive unbiased dataset tests.

A meaningful dataset with well-defined annotation of cancer driver gene sequences is gathered. The dataset is required as a benchmark of authentic cancer driver gene sequences. The benchmark dataset selected for this study is extracted from the most recent version available on the website namely IntOgen^12^. IntOgen lists in all 26,725 cases of mutations in a wide variety of human genes. Most of the cases are passenger mutations that do not cause cancer while 2901 listed mutations are tumor-causing. A total of 568 cancer driver genes are involved in these tumors causing mutations. Moreover, 1754 genes involved in passenger mutations were selected exhibiting the least mutual homology. Subsequently, data gathered in this way is used to formulate a benchmark dataset for the described problem. The benchmark dataset within the current study is denoted as G, which is defined as

1 $$G={G}^{+}U {G}^{-}$$

After subtle preprocessing and homology reduction a database was formulated, the final benchmark dataset contained 568 positive human gene sequences ${(G}^{+})$ and meticulously selected 1743 negative samples of negative gene sequences ${(G}^{-})$ obtained from a large collection of passenger genes.

### Sample formulation

A DNA sequence can be articulated as

2 $$S={\rho }_{1}, {\rho }_{2},{\rho }_{3},\dots ,{\rho }_{i}, \dots , {\rho }_{n}$$

where

3 $$\rho \in \{A\left(adenine\right),C\left(cytosine\right),G\left(guanine\right),T\left(thymine\right)\}$$

indicates the nucleotide at any arbitrary position, and $\in$ represents an image within the set hypothesis meaning “member of”^13^.

The recent advances in information and data sciences have furnished groundbreaking advances in biotechnology. However, one of the most pressing issues in devising such computational models that transform the raw data into discrete fixed-sized quantifiable models based upon their grouping information without losing any grouping physiognomies. Data and features obtained from such designs are instrumental in intelligent target analysis. Such vector detailings obtained exposure of genomic or proteomic arrangements are best appraised by machine algorithms (like ‘Neural Networks (CD)’, ‘Random Forest (RF)’ and ‘Support Vector Machine’ (SVM)) which are inherently designed to receive vector input^14^. It may well be conceivable that in a discrete model all the sequence-pattern data needs to be transformed into a fixed-size vector without losing crucial information that determines the properties of the given sequence. To overcome these constraints on sequence-pattern related data from proteins, the Pseudo Amino Acid Composition (PseAAC)^1^ was proposed. Later, Chou’s PseAAC^15,16^ has been deployed in about all of the computational proteomics arenas. Due to its ubiquity and significance in computational proteomics was incorporated into an efficient computer application called ‘PseAAC-General’^17^. Empowered by the triumphs of utilizing PseAAC to bargain with protein/peptide arrangements, its thought has been amplified to bargain with DNA/RNA arrangements in computational genomics via PseKNC (Pseudo K-tuple Nucleotide Composition)^13^. Subsequently, the genomic data is transformed into generalized stable numerical encoding depicted as *R* of Eq. (3) as

4 $$R=\left[ {\zeta }_{1}{\zeta }_{2}{\zeta }_{3}{\zeta }_{4}\dots {\zeta }_{u}\dots {\zeta }_{\Omega }\right]$$

where ${\zeta }_{v}$ (*v* = 1, 2,…,* Ω*) is an arbitrary numerical coefficient representing a feature. The components of Eq. (4) are useful data extricated from the gene sequence. Further, we discuss the methodology used to extract these features.

### Statistical moments

For characterizing the components and measurements of Eq. (4) and to have the quantitative portrayal for cancer driver gene samples of benchmarks dataset we utilize a factual approach. Statistical moments are applied to transform the genomic data into a fixed size. Each moment describes some unique information that designates the nature of data. Analysts and mathematicians have worked on moments of different distributions^18,19^. Hahn, raw and central moments of the genomic data are furnished into the feature set and forms a salient component of an input vector for the predictor. The area and scale of variance incorporated into the moments act as a tool to decipher among functionally different sequences. Moreover, other moments that define the asymmetry and the mean of data also help in the construction of a classifier with the appropriation of a labeled dataset. Scientists have observed that the properties of genomic and proteomic sequences are dependent upon the composition as well as the relative positioning of their bases. Henceforth, for furnishing the feature vector, only those mathematical and computational models are most apt which are sensitive to the relative positioning of component nucleotide bases within genomic sequences. It is a critical factor in formulating yielding and assiduous feature sets^18–26^. Since Hahn moments require two-dimensional data, therefore, the genomic sequences are converted into a two-dimensional notation *S’* of size k*k which stores the same information as *S* but in a two-dimensional form such that

$$k=\sqrt{n}$$

and

5 $${S}^{\mathrm{^{\prime}}}= \left[\begin{array}{ccc}{S}_{11}& {S}_{12}\dots & {S}_{1n}\\ {S}_{21}& {S}_{22}\dots & {S}_{2n}\\ \begin{array}{c}:\\ {S}_{n1}\end{array}& \begin{array}{c}:\\ {S}_{n2}\end{array}\dots & \begin{array}{c}:\\ {S}_{nn}\end{array}\end{array}\right]$$

Further, statistical moments are computed from the obtained square matrix for dimensionality reduction and forming fixed-size feature vectors^27,28^. As discussed previously, the three moments deployed in this study are Hahn, central and raw moments.

Equation 6 describes the operations performed to compute raw moments of order a + b.

6 $${U}_{ab}={\sum }_{e=1}^{n}{\sum }_{f=1}^{n}{e}^{a} {f}^{b} {\delta }_{ef}$$

Moments up to order 3 encompass significant information inscribed within the sequences, which are *U*_*00*_, *U*_*01*_, *U*_*10*_, *U*_*11*_, *U*_*20*_, *U*_*02*_, *U*_*21*_, *U*_*12*_, *U*_*03*_, and *U*_*30*_. Furthermore, to compute the central moments the centroid $(\overline{x },\overline{y })$ needs to be computed. The centroid acts as the center of data being visualized. Using this centroid the central moments are computed as:

7 $${V}_{ab}={\sum }_{e=1}^{n}{\sum }_{f=1}^{n}{\left(e-\overline{x }\right)}^{a} {\left(f-\overline{y }\right)}^{b} {\delta }_{ef}$$

The square grid is used as the discrete input to compute Hahn moments. Hahn moments help describe the symmetry of data and at the same time, they are reversible. This essentially means that these moments can be used to reconstruct the original data. The reversibility of moments ensures that the information curtailed within the original sequences remains intact and is passed forward to the predictor through the corresponding feature vector. Hahn moments are computed using Eq. (8).

8 $${h}_{n}^{x,y}\left(p,Q\right)={\left(Q+ V-1\right)}_{n}{\left(Q-1\right)}_{n}\times {\sum }_{z=0}^{n}{\left(-1\right)}^{z}\frac{{\left(-n\right)}_{z} {\left(-p\right)}_{z }{\left(2Q+x+y-n-1\right)}_{z} }{{\left(Q+y-1\right)}_{z }{\left(Q-1\right)}_{z }}\frac{1}{z!}$$

Equation (8) makes use of the Pochammer notation which in turn uses the Gamma operator, both these functions are elaborated by Akmal et al.^29,30^. Usually, the Hahn coefficient obtained in Eq. (8) is normalized using the coefficient described in Eq. (9).

9 $${H}_{pq}= {\sum }_{j=0}^{G-1}{\sum }_{i=0}^{G-1}{\delta }_{pq}{h}_{p}^{a,b} \left(j,Q\right){h}_{q}^{a,b}\left(i, Q\right),\quad m,n=0, 1, 2, \dots , Q-1$$

### Position relative incidence matrix (PRIM)

The proposed computational models are furnished for specific purposes only and are framed within the overall picture for prediction of gene attributes assigning them the essential grouping as a tool for readily identifying its remarkable traits. There is a clear quantization concerning the precise role of the nucleotide bases. Besides the bases, the position at which each base is placed is very significant. The position relative incidence matrix^31–33^ is introduced as an account of the relative positioning of nucleotide bases regarding each other. The organization of the matrix is given below:

10 $$R_{PRIM} = \left[ {\begin{array}{*{20}l} {R_{1 \to 1} } \hfill & {R_{1 \to 2} \ldots } \hfill & {R_{1 \to q} \ldots } \hfill & {R_{1 \to M} } \hfill \\ {R_{2 \to 1} } \hfill & {R_{2 \to 2} \ldots } \hfill & {R_{2 \to q} \ldots } \hfill & {R_{2 \to M} } \hfill \\ : \hfill & : \hfill & : \hfill & : \hfill \\ {R_{p \to 1} } \hfill & {R_{p \to 2} \ldots } \hfill & {R_{p \to q} \ldots } \hfill & {R_{p \to M} } \hfill \\ : \hfill & : \hfill & : \hfill & : \hfill \\ {R_{M \to 1} } \hfill & {R_{M \to 2} \ldots } \hfill & {R_{M \to q} \ldots } \hfill & {R_{M \to M} } \hfill \\ \end{array} } \right]{ }$$

In the above matrix, the aggregate of relative places of *q*^*th*^ base regarding the first occurrence of *p*^*th*^ base is represented in the component ${R}_{p\to q}$. The matrix hence obtained is further used to compute Hahn, central and raw moments and form coefficients of up to order 3.

### Reverse Position Relative Incidence Matrix (RPRIM)

The core objective of feature extraction is to uncover obscure patterns that are embedded within the gene sequences. The gene sequences need to be analyzed from varying perspectives to sieve out all the information pertinent to its behavior. Experiments show an analysis of the reversed sequence of gene/protein also reveals significant information. The reverse position relative incidence matrix (RPRIM) is formed based on exactly the described motive. This matrix is derived by reversing the original sequence and then computing the PRIM for the reversed sequence. An arbitrary element on RPRIM say ${R}_{\mathrm{i}\to \mathrm{j}}$ hold information regarding the relative positioning of the *ith* nucleotide base relative to the *jth* nucleotide base within the reverse sequence. The RPRIM matrix is represented as

11 $$R_{RPRIM} = \left[ {\begin{array}{*{20}l} {R_{1 \to 1} } \hfill & {R_{1 \to 2} \ldots } \hfill & {R_{1 \to q} \ldots } \hfill & {R_{1 \to M} } \hfill \\ {R_{2 \to 1} } \hfill & {R_{2 \to 2} \ldots } \hfill & {R_{2 \to q} \ldots } \hfill & {R_{2 \to M} } \hfill \\ : \hfill & : \hfill & : \hfill & : \hfill \\ {R_{p \to 1} } \hfill & {R_{p \to 2} \ldots } \hfill & {R_{p \to q} \ldots } \hfill & {R_{p \to M} } \hfill \\ : \hfill & : \hfill & : \hfill & : \hfill \\ {R_{M \to 1} } \hfill & {R_{M \to 2} \ldots } \hfill & {R_{M \to q} \ldots } \hfill & {R_{M \to M} } \hfill \\ \end{array} } \right]$$

Further for uniformity, the Hahn, raw, and central moments are also computed.

### Frequency vector determination

The sequence order, as well as the composition of a nucleotide chain, impart their effect in exhibiting the overall attributes of a gene. Previously, the PRIM and RPRIM matrices we discussed, both these matrices help to extract the sequence-related correlations of the nucleotide bases. The frequency vector (FV) summarizes the composition-related information of a gene. Each element of this vector provides the count of the occurrence of the nucleotide within the gene sequence. The vector is represented as

12 $$\alpha =\left\{{\varepsilon }_{1}, {\varepsilon }_{2}, \dots ,{\varepsilon }_{n}\right\}$$

where ${\varepsilon }_{i}$ provides the count of the overall occurrence of the *ith* nucleotide base.

### Accumulative absolute position incidence vector (AAPIV) generation

A feature extraction model that is capable of extracting all the aspects relevant to the sequence ordering and composition of the gene sequence. The FV provides information regarding the frequency of occurrence of each nucleotide base, similarly, the accumulative absolute position incidence vector provides the cumulative information regarding the position occurrence for each specific nucleotide base. It is represented as

13 $$K= \left\{{\lambda }_{1}, {\lambda }_{2}, \dots {\lambda }_{n}\right\}$$

where the *ith* component of AAPIV is computed as

14 $${\lambda }_{i }= {\sum }_{k=1}^{n}{\beta }_{k}$$

and ${\beta }_{k}$ is an arbitrary position of occurrence for a specific nucleotide. An arbitrary element ${\lambda }_{i}$ of AAPIV will contain the sum of all the positions of occurrence of the *ith* nucleotide.

### Reverse accumulative absolute position incidence vector (RAAPIV) generation

A deeper perspective regarding the hidden patterns within the gene sequence is provided by the reverse sequence. Computation of AAPIV for the reverse sequence of the gene is termed RAAPIV. It is represented as:

15 $$\curlywedge = \left\{ {n_{1} ,n_{2} , \ldots ,n_{m} } \right\}$$

An arbitrary element $n_{i}$ of RAAPIV will contain the sum of all the positions of occurrence of the *i*^*th*^ nucleotide within the reverse sequence.

### Feature vector formulation

**Figure 2:**
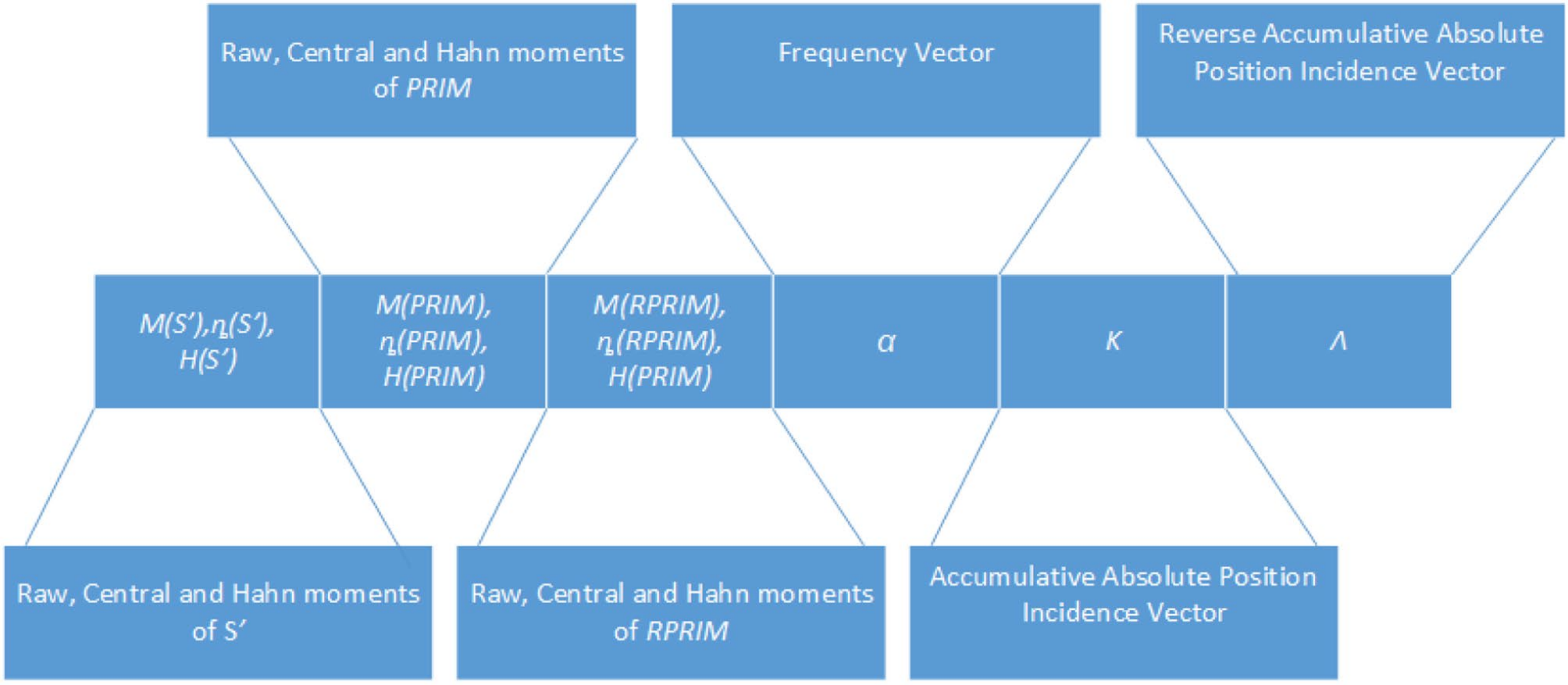
Depicts the structure of a feature vector.

Each primary sequence is transformed into a fixed scale notation of Eq. (4). Moments are computed Large matrices G’, PRIM, and RPRIM are transformed into succinct form by computing raw, central, and Hahn moments. Subsequently, these moments are assimilated into a feature vector along with FV, AAPIV, and RAAPIV. Figure 2. shows the structure of a feature vector illustrating each component. Coefficients contained in this feature vector corresponding to a primary sequence of arbitrary length. A comprehensive set of feature vectors is computed for all the samples in the dataset.

### Random forest

**Figure 3:**
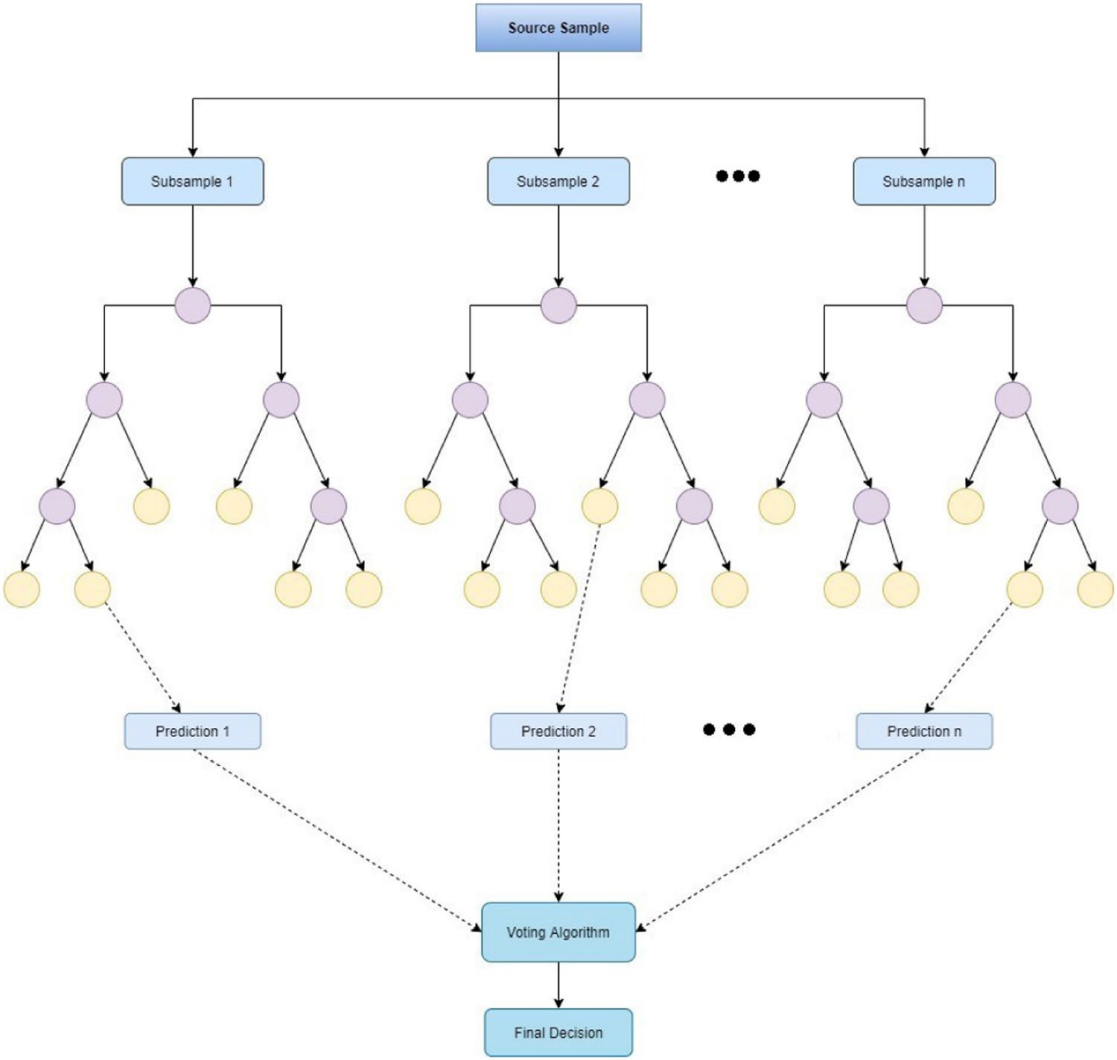
The architecture of random forest classifier for the proposed prediction model.

Random forests or random decision forests are an ensemble learning method for classification, regression, and other tasks that are performed by constructing a multitude of decision trees at training time. The overall output of the class is represented as the mode or mean of all the individual trees^34,35^. Figure 3 shows the architecture of the Random forest tree used for the purpose.

### Artificial neural network

**Figure 4:**
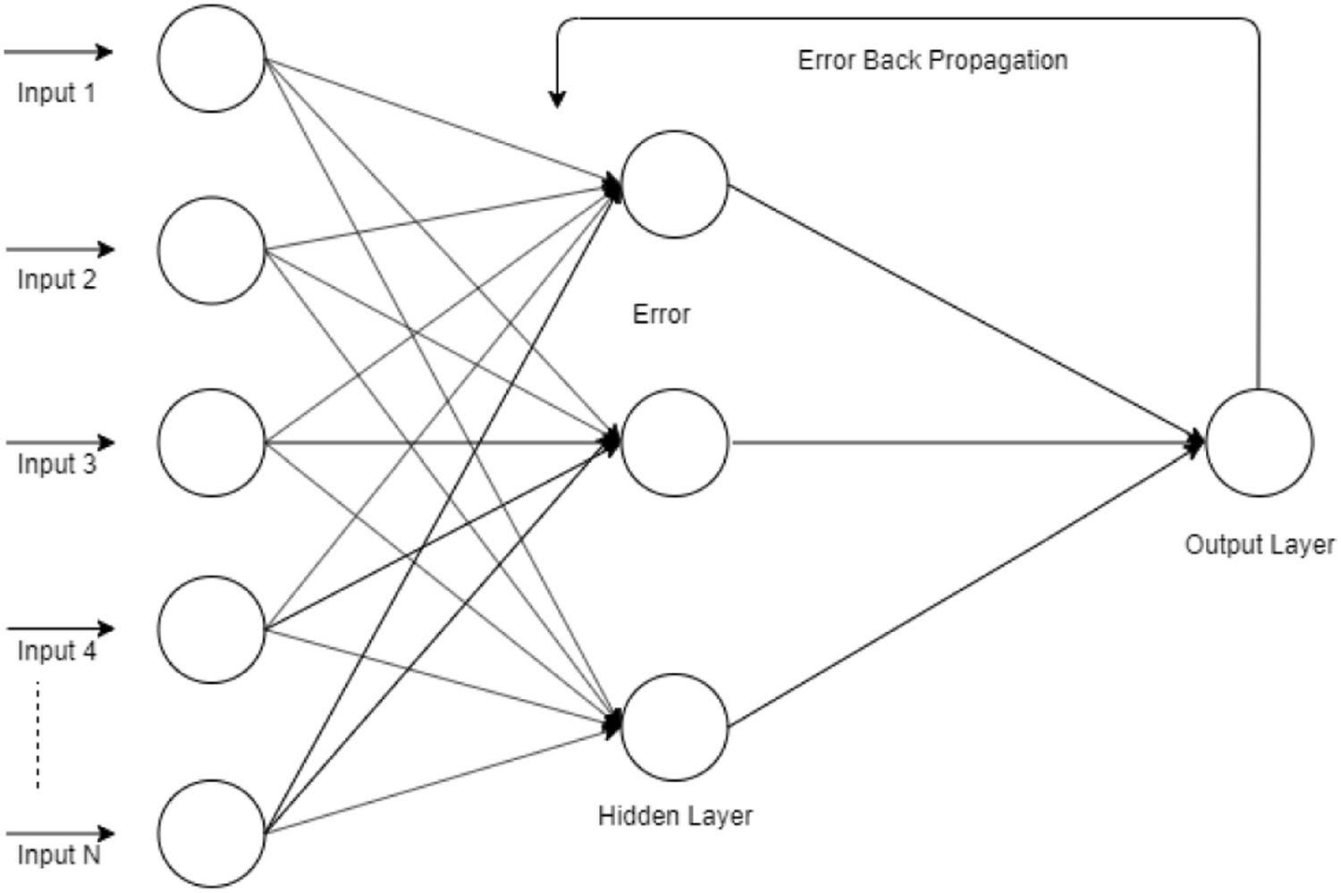
The architecture of the artificial neural network classifier for the proposed prediction model.

Artificial neural networks are a structure of linked neurons in which the output of the previous neuron in one layer is connected to the input of a neuron in the next layer. The activation function at each neuron uses the inputs from each neuron in the previous layer and their assigned weights to provide an output. The weights of neurons are adjusted iteratively until the objective function is achieved^24,25,34–36^. The architecture of the neural network used is described in Fig. 4.

### Support vector machine

**Figure 5:**
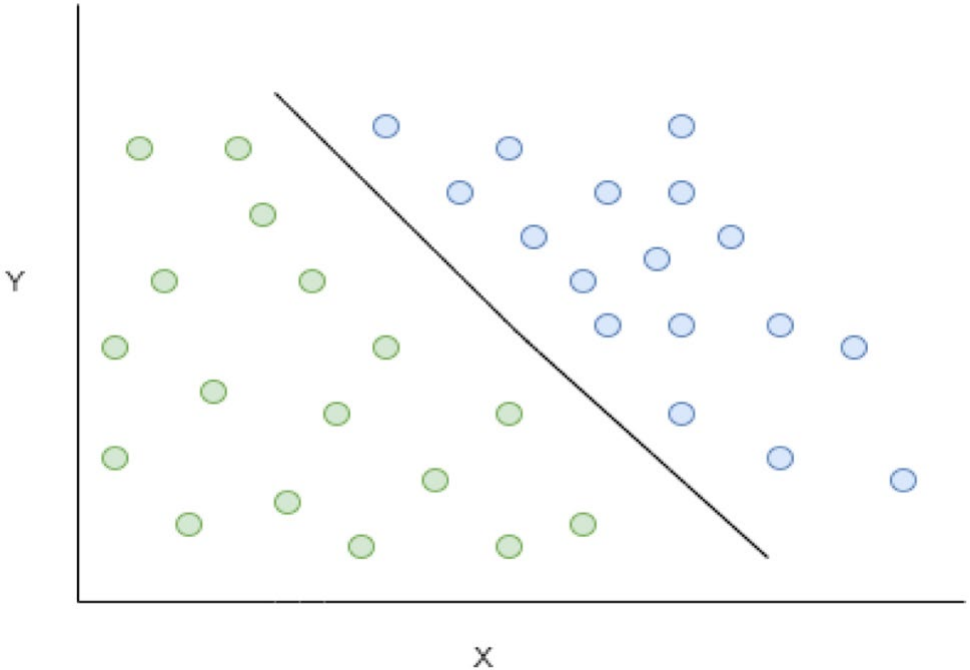
Architecture of SVM Classifier for the proposed prediction model.

Support-vector machines are supervised learning models with associated learning algorithms that analyze data used for classification and regression analysis. SVMs are commonly used in classification problems and as such. SVMs are based on the idea of finding a hyperactive plane, as depicted in Fig. 5 that best divides the dataset into two classes.

Support vectors are the data points nearest to the hyperplane, the points of a data set that, if removed, would alter the position of the dividing hyperplane. Hence, they are considered critical elements of a data set^37–39^.

Supervised learning models are those which require that the data is already annotated or labeled. On the other hand, unsupervised learning models use unlabeled data and they try to make sense of data as they learn patterns within it. Usually, classification problems in proteomics and genomics using experimentally obtained data regarding a specific phenomenon. Several proteomic and genomic databases are available which work as a repository of previous experimental findings. Known and well-annotated data is curated from such repositories. Hence, supervised learning models are the natural choice for classifiers using such data. Discussed supervised learning models are facilitated by combining all the feature vectors such that each row corresponds to a single primary sequence sample to form an input array. Since the data is experimentally collected and well-annotated, an expected output matrix is also furnished to facilitate supervised learning. Further, both these matrices are used to train the described classifiers. The input array is used as training data by these models while the expected outputs are used to calculate errors throughout the learning process of each model.

## Results and discussions

The estimation of the performance of the predictor in terms of accuracy is very important to ensure that the right kind of assessment for the prediction algorithm. To establish these facts researchers have developed several quantitative metrics. These metrics are established based on well-defined experimental tests and hence be confidently used as a comparative parameter among competitive models.

### Accuracy metrics

Four interlinked quantitative metrics are generally used to assess the performance of a predictor. The accuracy metric is denoted as Acc, it provides an overall picture of the prediction accuracy of the model. The second metric is named sensitivity denoted as (S_n_), which represents the capability of the model in accurately predicting positive samples. Similarly, the specificity denoted as (S_p_) is used to provide a quantitative measure for the accuracy of the model in predicting negative samples^40^. Lastly, Mathew’s correlation coefficient factor denoted as (MCC) is a stable measure for the accuracy of the predictor when the number of positive and negative samples is unbalanced.

For simplicity and readability, a formulation for these metrics introduced by W. Chen, Feng, & Lin in 2013^41^ is illustrated. The authors represented these metrics formulation expressions in such a way that they are more readable and easier to implement.

16 $${S}_{p}=1-\frac{{P}^{\pm }}{{P}^{-}} 0\le {S}_{p}\le 1$$

17 $${S}_{n}=1-\frac{{P}^{\mp }}{{P}^{+}} 0\le {S}_{n} \le 1$$

18 $$Acc=1-\frac{{P}_{- }^{+}+ {P}_{+}^{-} }{{P}^{+}+ {P}^{-} } 0\le Acc\le 1$$

19 $$MCC= \frac{1-\left(\frac{{P}^{\pm }}{{P}^{+}}+\frac{{P}^{\mp }}{{P}^{-}}\right)}{\sqrt{\left(1+\frac{{P}^{\mp }-{P}^{\pm }}{{P}^{+}}\right) \left(1+\frac{{P}^{\pm } {- P}^{\mp }}{{P}^{-}}\right)}}$$

where ${P}^{+}$ represents the actual number of cancer driver genes, ${P}_{-}^{+}$ is the number of cancer driver genes predicted as passenger genes, ${P}^{-}$ is the actual number of passenger genes in the test while ${P}_{+}^{-}$ is the number of passenger genes predicted as cancer driver genes. The above equations signify that the sensitivity Sn will be maximum when no sample is wrongly predicted as a passenger gene i.e. ${P}_{-}^{+}=0$, similarly, the sensitivity Sp is maximum when ${P}_{+}^{-}=0$. The overall picture of the predictor performance is portrayed by Acc and MCC. The prediction model is most accurate if Acc = 1 and also MCC = 1 which essentially means that no sample has been wrongfully predicted i.e. ${N}_{-}^{+}= {N}_{+}^{-}=0$. There are some other changes as well in which it is possible that no solitary cancer driver gene gives you the positive dataset and all the non-cancer driver genes give you the negative dataset and show that the figures are dishonestly anticipated by the researchers. In the case of the other extreme, MCC value will be − 1 and the value of Acc will be 0. In the case of a binary predictor, there are only two classes and the probability of predicting correctly is 50%. An accuracy of 50% is considered as a benchmark, any predictor to be acceptable must at least have an accuracy better than 50 percent. For such a predictor the value of MCC will be 0 and the value of Acc will be 0.5 where ${N}_{+}^{-}={N}^{-}/ 2$ and ${N}_{-}^{+}={N}^{+}/ 2,$ which essentially implies that only half of the cancer genes and half of the passenger genes will be predicted accurately. The use of these metrics brings acceptability and acclaim to the experimental results established by the study. Furthermore, these metrics can also be expanded for multiclass predictors^7,42–44^. The performance of a predictor is substantiated through a set of well-defined tests. These tests yield accuracy metrics which firstly signify quantitatively how well a model performs and secondly, establishes the suitability of the model even if new data is not readily available.

### Self-consistency validation

**Figure 6:**
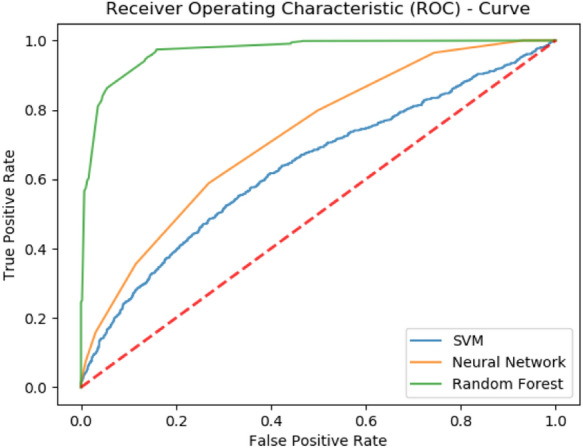
Self-consistency ROC curves for PCDG-Pred.

The self-consistency test is one of the most basic tests and is generally used to establish the appropriateness of the predictor. Previously, a set of feature vectors constituting positive and negative samples was collected and used for training. After sufficient training of the model, it is validated using a self-consistency test. This test reverberates that the predictor is tested with the same samples which were used to train it. Henceforth, all the classifiers trained on the benchmark dataset are tested. The number of samples correctly predicted by each of the classifiers is tabulated to calculate the accuracy metrics as shown in Table 1. Consequently, a receiver operating characteristic (ROC) curve shows a comparison of accuracy exhibited by each predictor. It can be noticed that the performance of the Random Forest-based predictor is fairly thriving as compared to SVM and neural network-based predictor. In Fig. 6, it is noticed that the area under the curve of the RF-based predictor is maximum.

**Table 1:** Self-consistency experimental results for PCDG-Pred.

Method	Accuracy metrics			
	Sn (%)	Sp (%)	Acc (%)	MCC (%)
PCDG-RF	88.10	92.26	91.08	0.7867
PCGD-SVM	50.16	72.14	69.45	0.1585
PCGD-NN	64.71	77.75	75.19	0.3660

All the results yielded by the described test are shown in Table 1 and Fig. 1, as illustrated. It shows that the predicted rule which was performed during the study was similar to the original proposed computational method of the study. It also shows the normal execution of all the proposed frameworks which are concerned with this research and the study.

### Independent set testing

Independent set testing is the most trivial test for gauging the functioning conduct of the predictor for unknown data. Usually, to perform this test the data is partitioned into two unequal-sized partitions. The larger partition is used to train the predictor and the smaller one is used to test its accuracy. Since numerous permutations exist for making such partitions, the test is repeated several times to ascertain its accuracy. The extracted benchmark dataset is used to perform this test. A partition spanning over 70% of the dataset was used to train the predictors, while the rest of the 30% was left for testing. The test was repeated 10 times with different partitions. The average accuracy metrics obtained from each predictor are listed in Table 2.

**Table 2:** Independent testing results for PCDG-RF.

Method	Accuracy metrics			
	Sn (%)	Sp (%)	Acc (%)	MCC (%)
PCDG-RF	91.06	81.27	87.26	0.6482
PCGD-SVM	48.21	68.11	65.42	0.412
PCGD-NN	61.67	74.36	71.87	0.331

**Figure 7:**
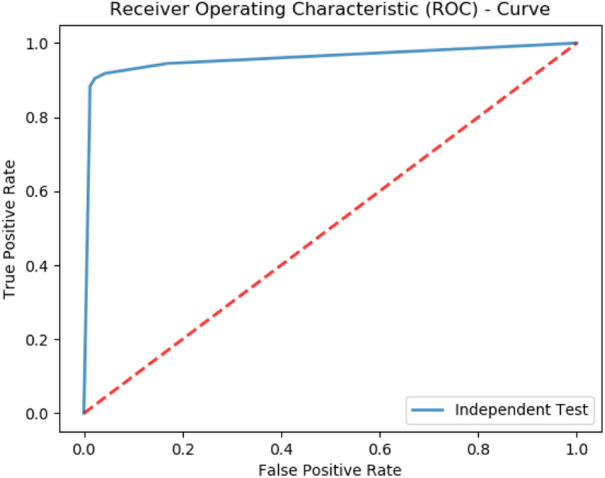
Independent Set testing for PCDG-RF.

Again, the test shows that the RF-based predictor outperforms the accuracy of SVM and neural network-based predictors. The comparison of all the machine learning models is shown in the ROC graph in Fig. 7.

### Cross-validation

The self-consistency test provides some feedback regarding the comparative performance of different models. However, it does not reflect the capability of the model to work on unknown data. Independent data set testing does provide a notion regarding the capability of the model to work on unknown data. Although, independent set testing is performed on randomly partitioned data still there is still a possibility that a major troublesome portion of data is left out of this test. A more rigorous test that alleviates this situation is the cross-validation test. Cross-validation is a rigorous test that spans over all the samples^32,33,42,45^. The dataset is partitioned into *k* disjoint folds. The test will be repeated k times. Each time the testing is performed on a different partition while the rest of the *k-1* partitions are used for training. In the end, the results reported from each test carried out *k-*times are averaged to compute the overall prediction accuracy. This strategy proves most beneficial when new test data is not readily available. The cross-validation test provides a fair picture regarding the overall performance of the predictor on unknown data as no data item is left out of the testing process. The cross-validation is a rigorous test as it spans over all the datasets and every item of the dataset has to be tested on the prediction model. tenfold cross-validation is performed on all the predictors. The data is divided into 10 disjoint sets such that their composition forms the whole data. Items within each dataset are placed by randomly selecting them from the combined dataset. Each fold is tested on predictors furnished through training of the rest of the folds. The average accuracy metrics obtained from testing of all 10 folds are reported in Table 3 for all the predictors.

**Table 3:** Tenfold cross-validation results for PCDG-Pred.

Method	Accuracy metrics			
	Sn (%)	Sp (%)	Acc (%)	MCC (%)
PCDG-RF	84.12	96.12	92.48	0.8141
PCGD-SVM	71.66	99.54	86.81	0.552
PCGD-NN	81.39	83.01	88.23	0.601

**Figure 8:**
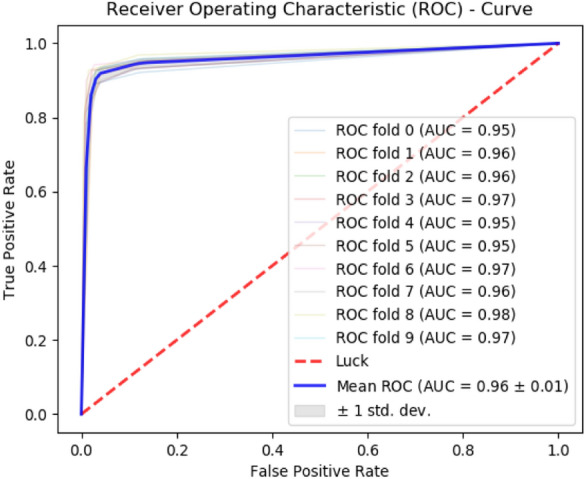
10-Fold cross-validation for all predictors.

Subsequently, Fig. 8 shows a ROC curve depicting the performance of the most optimal PCDG-RF predictor. This test also establishes that the RF-based algorithm provides greater yield as compared to SVM and neural network.

**Figure 9:**
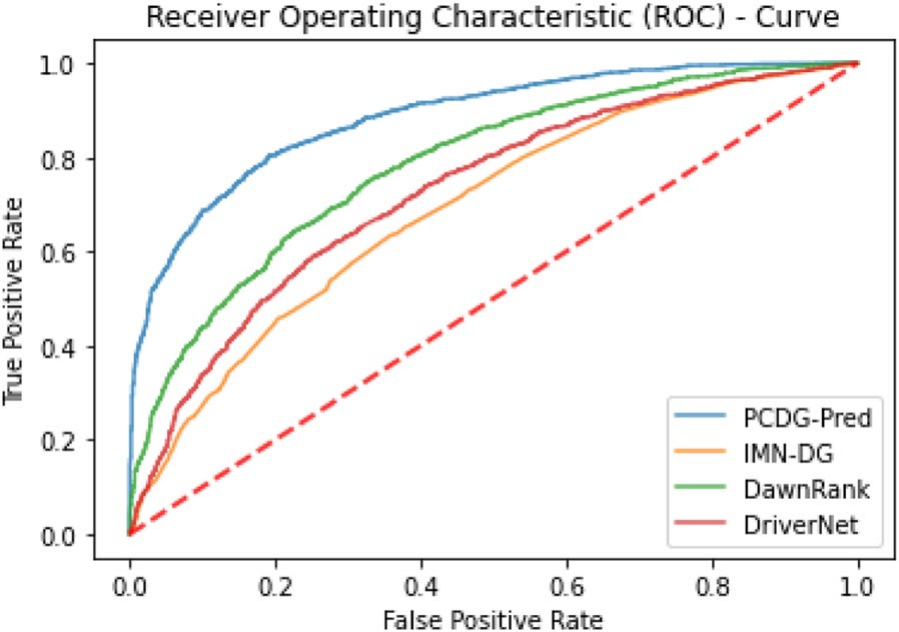
Shows ROC curve depicting the performance of proposed predictor along with existing predictors. The area under the curve (AUC = 0.84) for the proposed technique promises the best performance.

Randomly curated cancer driver samples over numerous iterations were tested on PCDG-Pred along with other existing cancer driver gene predictors namely DawnRank^46^, DriverNet^47^, and IMN-DG^48^. The DawnRank algorithm ranks a gene based on the disruption it causes resulting in downstream genes. DriverNet studies the disruptions caused in transcriptional patterns due to genomic aberrations leading to cancer. Integrative Module-Based Cancer Driver (IMN-DG) prediction methodology proposes machine learning techniques for the identification of cancer driver genes. The comparison of these techniques is illustrated in ROC curves (Fig. 9) plotted using the mean of True Positive Rate (TPR) and False Positive Rate (FPR) for all the iterations.

The graph shows that the proposed technique outperforms the previous technique as it shows the greatest area under the curve (AUC) of 0.84 for the curated datasets.

Identification of cancer driver genes is an important aspect of precision and personalized oncology. Bioinformatics tools that could accurately and readily analyze sequencing data in this respect have insightful demand. The proposed model is systematically organized by accumulating robust data, applying meaningful feature extraction techniques, and then using state of art machine learning algorithms for training. Further, the model is rigorously tested and validated using different validation techniques. Test results exhibit that the PCDG-RF has the best accuracy of 91.08%, 87.26%, and 92.48% for self-consistency, independent set testing, and tenfold cross-validation respectively. The other algorithms were unable to match PCDG-RF which yielded an accuracy for tenfold cross-validation of 86.81% and 88.23% for PCDG-SVM and PCDG-NN respectively. Further, to ascertain the robustness of the proposed model, its accuracy was tested against other existing models for the identification of cancer drivers. A randomly selected set of cancer driver genes was iteratively tested with the proposed model along with existing models such as DawnRank, DriverNet, and IMN-DG. Results hence obtained are illustrated by plotting a receiver operating characteristics graph using the true positive rate and false-positive rate. The proposed model performs significantly better as compared with existing models with an area under the curve of 0.84 while others seem to cover a lesser area as illustrated in Fig. 9. Based on these findings it can be confidently concluded that the proposed model can be used as a robust tool for the identification of cancer driver genes while playing an important role as a bioinformatics tool in cancer research.

### Web server

The significance of a web server application is thoroughly realized by the fact that it has all the abilities to provide an easy-to-understand computational analysis promptly. This need is felt by leading researchers working in a specific domain. Researchers working on such studies try to make sure that the webserver is freely available to help any future developments and advancements relevant to the study. Furthermore, the whole picture implies the fact that the fundamentals of computational science have improved in the last few years especially those relevant to data science and classification^31^. This further strengthens the notion that computational applications assisting medical science are going for an upheaval^32^ shortly. For all these reasons several efforts are made to make sure that a web server is developed for all the prediction techniques used these days. Thus, a web-based implementation of the methodology has been made available at https://pcdg-pred.herokuapp.com.

## Conclusion

Oncology is recognized as a highly prioritized area of research. Science and research have uncovered substantial myths regarding cancer by identifying well-defined targets to investigate and mediate improvements in this area. Researchers have established that cancer is a genetic disorder. Cancer is driven by distinctive changes and basic variations in a genetic structure called mutations. In case a gene mutation results in uncontrollable growth of cancer then the gene is called the cancer driver gene otherwise, if the mutation does not cause cancer then it’s called a passenger mutation. Cancer is one of the most preeminent disorders whose progression has nefarious effects on human life and may eventually lead to death. Recognition of cancer driver genes is a basic instrument of oncological studies. Numerous methods exist for the identification of oncological genetic progression through mutation if provided with years old original sequence along with the current mutated one. Researchers are actively working to provide personalized treatment for this life-threatening disorder. Gene silencing is a key area being inquired by researchers that could help silence specific cancer driver genes. The proposed bioinformatics tool can greatly facilitate researchers and the medical community in this aspect by identification of specific cancer driver gene sequencing obtained through next-generation sequencing of patient DNA. This paper uses different techniques and devices a feature extraction technique based on the relative positioning of nucleotide bases. Subsequently, for dimensionality reduction, statistical moments are employed to form a fixed-sized feature vector for each gene sequence. The most recent set up to date data regarding cancer drivers and passenger genes is extracted from IntOgen site. The feature set formed is used as input for the training of the various classifiers. Experimental results show that the most suitable and accurate classifier for deciphering obscure patterns among cancer driver genes is random forest. Hence, a combination of devised feature sets and random forest forms a comprehensive platform for the identification of cancer driver genes. The best accuracy achieved for the tenfold cross-validation test is 92.48%. The present study can indeed aid in the emerging personalized treatment of cancer through the identification of specific cancer-causing genes using DNA sequencing of the patient.

## Supplementary Information

Supplementary Information 1.

Supplementary Information 2.

Supplementary Legends.
