# Supplementary material for: Evaluating machine learning methodologies for identification of cancer driver genes
Source: Sci Rep. 2021 Jun 10;11:12281. doi: 10.1038/s41598-021-91656-8 (PMC8192921; doi:10.1038/s41598-021-91656-8)
Supplement: Supplementary file 3 — Supplementary Legends. [file 41598_2021_91656_MOESM3_ESM.docx]

**Supplementary Material**

Supplementary File 1 contains the sequence of the positive samples of cancer driver genes.

Supplementary file 2 contains sequence of negative samples (genes in which cancer driver mutations are not observed).
